# Classifying forensically important flies using deep learning to support pathologists and rescue teams during forensic investigations

**DOI:** 10.1371/journal.pone.0314533

**Published:** 2024-12-05

**Authors:** Anna Katharina Gohe, Marius Johann Kottek, Ricardo Buettner, Pascal Penava

**Affiliations:** Chair of Hybrid Intelligence, Helmut-Schmidt-University / University of the Federal Armes Forces Hamburg, Hamburg, Germany; Universidade Estadual de Feira de Santana, BRAZIL

## Abstract

Forensic entomology can help estimate the postmortem interval in criminal investigations. In particular, forensically important fly species that can be found on a body and in its environment at various times after death provide valuable information. However, the current method for identifying fly species is labor intensive, expensive, and may become more serious in view of a shortage of specialists. In this paper, we propose the use of computer vision and deep learning to classify adult flies according to three different families, *Calliphoridae, Sarcophagidae, Rhiniidae*, and their corresponding genera *Chrysomya, Lucilia, Sarcophaga, Rhiniinae*, and *Stomorhina*, which can lead to efficient and accurate estimation of time of death, for example, with the use of camera-equipped drones. The development of such a deep learning model for adult flies may be particularly useful in crisis situations, such as natural disasters and wars, when people disappear. In these cases drones can be used for searching large areas. In this study, two models were evaluated using transfer learning with MobileNetV3-Large and VGG19. Both models achieved a very high accuracy of 99.39% and 99.79%. In terms of inference time, the MobileNetV3-Large model was faster with an average time per step of 1.036 seconds than the VGG19 model, which took 2.066 seconds per step. Overall, the results highlight the potential of deep learning models for the classification of fly species in forensic entomology and search and rescue operations.

## Introduction

Each year, forensic examinations are required for approximately 464,000 people who were victims of intentional homicide [1]. For economic, psychological, and social reasons, finding the murderer quickly in these cases is essential [2–4]. In this context, estimating the time of death is one of the most important success factors [5, 6].

As the use of natural methods in determining the time of death is characterized by growing inaccuracy with increasing time interval after death, forensic entomology has emerged [6–8]. In this context, one possibility is the identification of fly species, such as *Calliphoridae, Sarcophagidae*, and *Rhiniidae*, that can be found on and around the dead body at different times after death, allowing closer conclusions to be drawn about the time of death [9–11]. Currently, however, systematic identification of fly species is based on human evaluation, which is labor-intensive and expensive, and may further be complicated by the lack of experts [11–13].

A possible solution to this lack of efficiency are computer vision and deep learning [14, 15]. Thanks to cameras and sensors, entomological observations of flying insects can be made continuously and without the presence of a human being. Until now laser technology adapted to entomology, distance sensors and radar are used [16]. Deep learning models can be trained with the received data and indicate the species of insects and their abundance [17].The prediction with deep learning differs from conventional statistical approaches [18] because deep learning models learn without external feature specifications, but rather by recognizing features independently through a variety of iterations [19]. In this context, computer vision and deep learning represent one way to mitigate this challenge [11].

However, so far, such a digital application has only been developed for the analysis of maggots [20]. However, in this context, it should be noted that *Sarcophagidae* in particular, which is one of the most prevalent fly species in forensics, cannot usually be determined from the morphological analysis of the larvae [8, 12]. Furthermore, the successful use of entomology depends on the time at which the evidence samples were extracted [21]. The storage of the corpse as well as the different temperatures, for example in the body bag, allow for a changed development of the maggots compared to the crime scene itself, leading to different results with respect to the time of death [22]. Depending on the season and other abiotic factors, blowflies such as some *Calliphoridae* species can also lay eggs prematurely, so that the post-mortem interval (PMI)-estimation does not always agree completely with the literature [21].

Not only the change in living evidence, but also the fact that time management for the entire crime scene investigation is a bottleneck in finding all the necessary evidence are reasons for the deployment of drones [23]. With the help of drones, data of the flies around the body can be collected as soon as a body is found, whereas the sampling and examination of maggots usually begins at the autopsy after transportation [22]. It can be concluded that with a snapshot of the crime scene, estimating the PMI with the help of forensically important flies can have decisive advantages.

**Fig 1:**
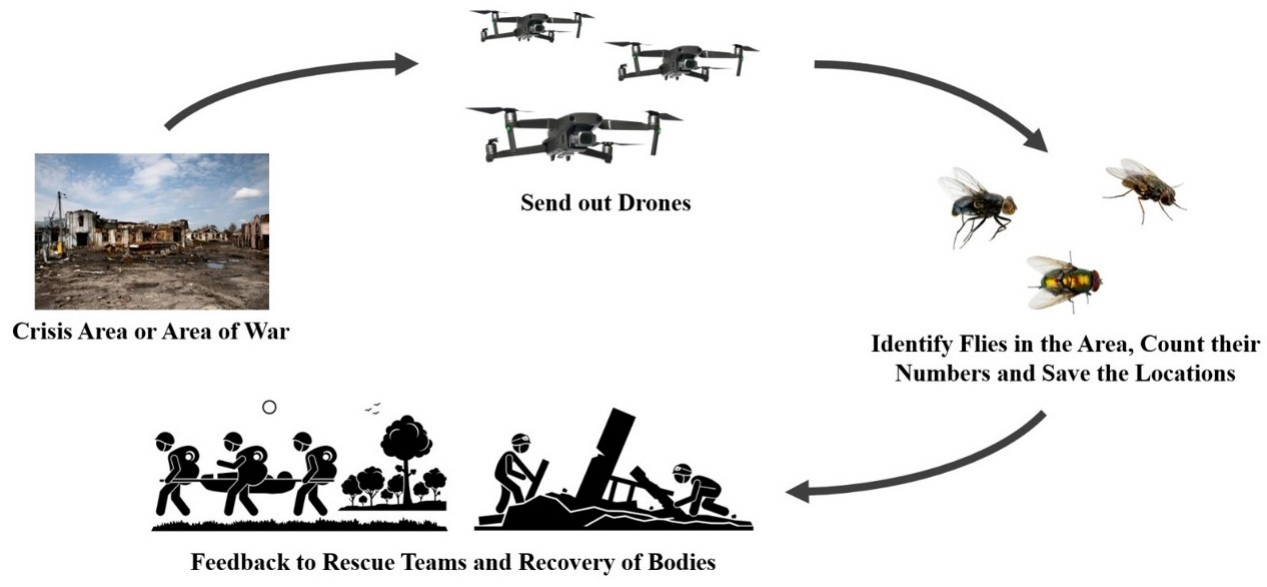
Use case: Finding bodies in crises areas by classifying forensic flies using a drone.

In addition especially in a time of wars and natural disasters when people disappear, for example by being buried, current methods need national imperative and resources, which on the one hand show the evolution and, on the other hand the effort that is still associated with the rescue [24]. As illustrated in (Fig 1) Drones that scan the crises areas for flies can help search for bodies or even find living people lying next to those who have already died [23]. Therefore, the determination of adult flies could be crucial [12, 25], but so far there is no deep learning tool.

Therefore, the aim of this work is to develop a deep learning model that can classify adult flies according to the three different families *Calliphoridae, Sarcophagidae, Rhiniidae* and their corresponding genera *Chrysomya, Lucilia, Sarcophaga, Rhiniinae* and *Stomorhina* to improve the efficiency of PMI estimations in forensic investigations, especially in areas that are difficult to access or in large areas of crises that are impossible to search by hand. This paper presents an initial solution for the use of drones in forensic investigations and body searches.

Our most important contributions are as follows:

1. We built two machine learning models that classify the different flies with an excellent average accuracy of over 99%.
2. We trained a model using MobileNetV3-Large to enable mobile use and thus take an initial step toward the use of drones as a tool for the classification and detection of flies.
3. The model is fast and allows for a more cost-effective diagnosis that counteracts the shortage of skilled workers.
4. Connecting our model for the classification of flies in difficult areas and the model of Apasrawirote et al. [20] for the identification of larvae during autopsy can offer a promising advantage in forensic investigations.

This paper presents the research background on the concept of forensic entomology, as well as related work in the next section. Then the methodology used to build the machine learning model for the classification of forensically important flies, including the training process, is explained. Next, the results section explains the performance of the models with the help of performance indicators and a confusion matrix. In addition, an in-depth discussion of the findings and their implications follows afterwards. Finally, the conclusion shows all findings, as well as the limitations of the work and potential future research and development.

## Research background

### Concept of forensic entomology

Forensic entomology finds its beginning in the 13th century China. After a farmer had been murdered with a sharp weapon and no evidence was found visible to the human eye, investigators caught the murderer by observing the suspects’ sickles. Only the murderers sickle was surrounded by flies and so the case was solved [26]. The scientific adoption of insects as evidence can be traced back to a crime in France in the middle of the 19th century when a child was found behind a chimney. Based on the flies, it was concluded that the current residents of the house could not be held responsible for the death. This was the first time evidence based on forensic entomology was allowed in a courtroom [27].

Forensic entomology deals with the use of insects in criminal investigations and is applicable long after the death of the human being [8]. The process of postmortem decomposition is complex. The body is constantly changing, especially in the first hours and days of death. The longer death progresses, the more difficult it becomes to predict the PMI because the decomposition sets in and changes are more difficult to detect [28]. This is where forensic entomology becomes important. Insects are found in corpses at different times, some insects immediately after the onset of death, others only after months [29, 30]. The most important for forensic entomology are flies and beetles, [31] which belong to carrion eating, as well as predators and parasites species [30].

According to a study by Wolff et al., there are five major decomposition stages and four insect categories present during decomposition. Decomposition stages include fresh, bloated, active decay, advanced decay and dry remains. Insects can be divided into necrophagous, predators, omnivores, and incidental catch. In the order of fly the family of *Calliphoridae* with its genus *Chrysomya* can already be found in the bloated stage, which rests for 2-6 days. However, if a body has been lying for 52 days, no fly of the *Calliphoridae* family will be found. In comparison, *Sarcophagidae* will not be around a dead body until the active decomposition stage sets in. These flies will also be around until the end of the dry stage [25].

### Machine learning

Due to a huge amount of data, machine learning and with it deep learning has become a big part of artificial intelligence and is already utilized in many industries [32]. There are different types of network architectures, which are sometimes more or less suited for an application depending on the task. Artificial Neural Networks (ANN)[33], Convolutional Neural Networks (CNN) [34, 35] and k-nearest neighbors (KNN) [36] are examples for common machine learning algorithms. Deep learning algorithms, currently mainly CNN are used for data processing and for the evaluation of digital images [35].The main reasons for the use of deep learning models are that they achieve better results than traditional systems, they allow data augmentation which improves generalization and deep learning models that are light-weight also perform on devices with lacking hardware resources [37].

### Related work

Machine Learning has been used in different cases to identify maggots or fruit flies. An overview is provided in Table 1.

**Table 1:** Related work.

Author	Year	Classification Forensically important Maggots	Other Fruit Flies	Algorithm					Accuracy average
				CNN	RF	SVM	ANN	KNN	
Apasrawirote et al. [20]	2022	x		x					97.33%
Salifu et al. [38]	2022		x		x				91.10%
Salifu et al. [38]	2022		x			x			96.00%
Salifu et al. [38]	2022		x				x		96.00%
Salifu et al. [38]	2022		x					x	93.20%

Apasrawirote et al. [20] build a machine learning model for the classification of forensically important fly maggots. For this, they used data from a digital camera that was connected to a compound microscope. They applied different CNN model architectures to compare the results, but in the end they focused on the AlexNet model. On average the tested model reached an accuracy of 97.33%, with AlexNet achieving the best balance between power and speed.

In contrast to this Salifu et al. [38] used machine learning to analyze fruit fly morphometrics. They used secondary data on wing veins and tibia length of male samples collected from different parts of Africa and Asia. For machine learning, they tried four classifiers, K-nearest neighbor (KNN), random forest (RF), support vector machine (SVM) and artificial neural network (ANNs). This contribution showed that SVM and ANN-models reached the best results in classifying fruit flies.

The results of the different classification models showed that it is possible to reach high level accuracy for this type of image dataset with machine learning and that with CNN the best results were obtained. At the same time, it becomes clear that the current possibilities for the classification of important forensic flies are limited by the fact that there are no machine learning models. Therefore, new approaches are essential to improve the current situation described during autopsy and already during the search for bodies.

## Methodology

Advanced machine learning algorithms were used to achieve the recognition of different species of fly. The first section gives a brief theoretical introduction to CNN and the transfer learning technique, which was utilized for this paper. A description of the dataset is given in the following section, before the process of data augmentation and pre-processing is explained afterwards. In the forth section the architectures of the two models programmed for this image recognition task are exemplified. Finally, the model evaluation is described.

### Convolutional neural network and transfer learning

The use of convectional networks started already in the early 1990s, first being used for tasks like speech recognition and document reading. CNNs are engineered to process data that comes in the form of multiple arrays, such as color images [39]. They are inspired by the neurological process in animals and have a wide application field in image and video recognition, recommend systems, and natural language processing [40]. The architecture of typical CNNs consists of convolutional layers with pointwise non-linearity such as ReLU applied at the end and pooling layers. The convolutional layers detect local conjunctions of features, and the pooling layers merge similar features into one [39]. In order to obtain a higher representation of the original image, the outputs of these collections are tiled so that their input regions overlap [40]. In the case of images, local combinations of edges form motifs which assemble into parts, and parts become objects. This way convolutional neural networks can transform data, like the images in the dataset used in this paper, into high-level representations, making detection or classification possible [41].

The models presented in this paper utilise transfer learning through pre-trained models to solve the classification problem. Traditionally, machine learning algorithms address tasks in isolation. By developing methods for transferring information from one or more source tasks to a related target task, transfer learning aims to make machine learning as efficient as human learning [42]. This can be achieved using different techniques [42, 43], this paper uses models that were previously trained on the ImageNet classification dataset and inherit their weights and biases from this. These base models are built into our models and trained on the new dataset. This technique has been shown to improve the performance of CNNs [43].

### Dataset

**Fig 2:**
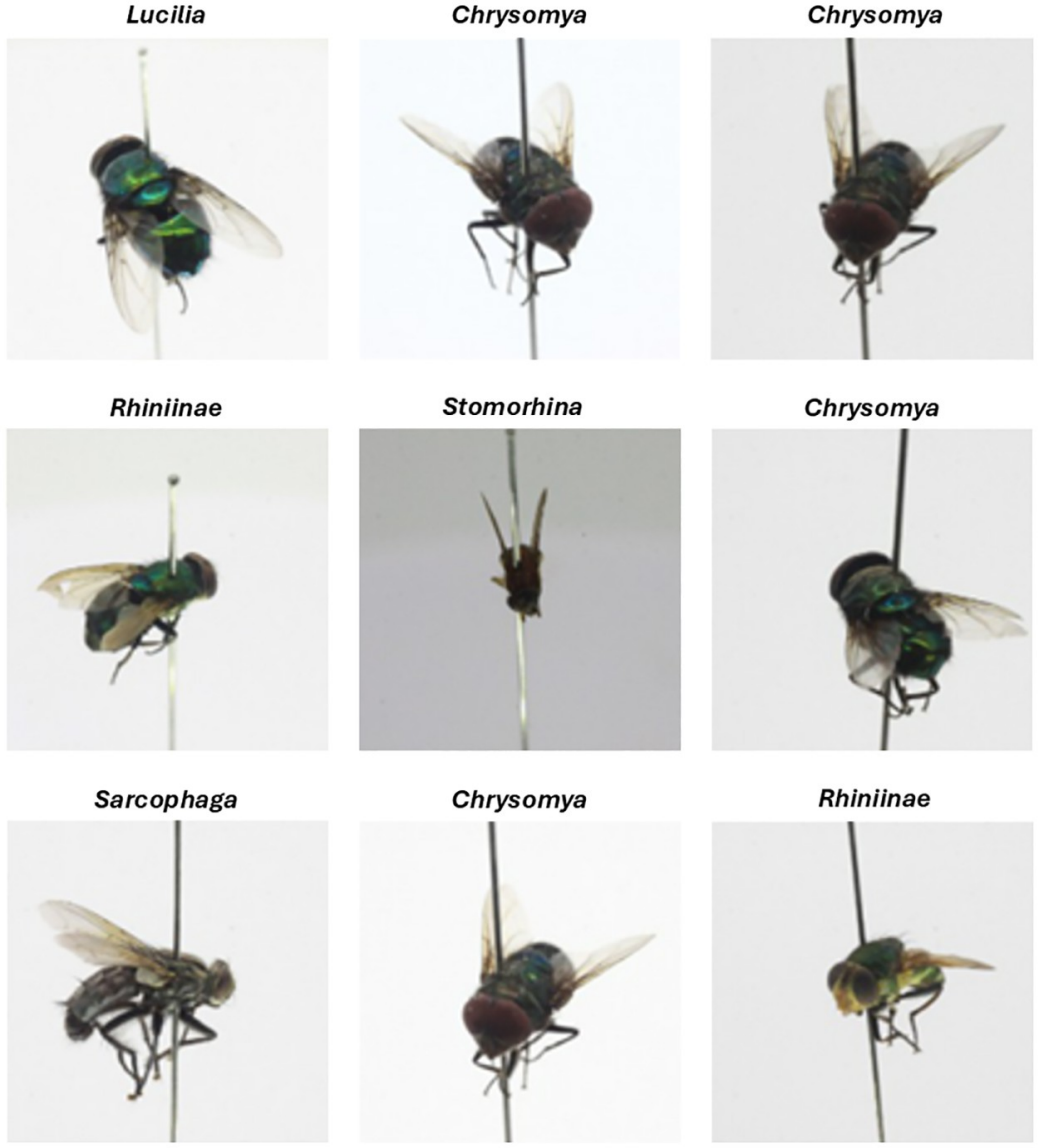
Exemplary images of the dataset.

The dataset came from the paper ‘An annotated image dataset of medically and forensically important flies for deep learning model training’ by Ong and Ahmad [11]. The dataset contains 2,876 images of five different fly genera, provided in 224 x 224 pixels and 96 x 96 pixels. The exact number of images per genera can be found in Table 2. For data collection, the researchers took insect specimens from the insect collection room of Borneensis, Institute for Tropical Biology and Conservation (ITBC), University Malaysia Sabah (UMS), and the School of Biological Sciences, University Sains Malaysia (USM). The insects were preserved in a room with 18°C and a humidity of 40+-5%. To photograph the flies a photography light box with dimensions of 30 x 30 x 30 cm and a digital single-lens reflex camera (Canon EOS 50D, 15.0 MP APS-C CMOS sensor) was used. The photos were taken from two different heights, a superior and a lateral position, and a 360° view. Due to the fact that blurry and poor quality images were removed, the data set provides an unbalanced number of images for each genus [11]. Fig 2.

**Table 2:** Number of images for each fly genera.

Fly Genera	Number of Images
*Rhiniinae*	488
*Stomorhina*	500
*Sarcophaga*	570
*Lucilia*	586
*Chrysomya*	731

### Data augmentation and preprocessing

Around 2000 images are not enough to train most modern deep learning architectures, necessitating data augmentation. This creates artificial samples by altering the pictures, resulting in a more plentiful and richer dataset, which will in turn help train a more stable model and in part prevent overfitting [44]. We used several data augmentation techniques included in the tensorflow command “ImageDataGenerator”. Inputs were randomly flipped horizontally, rotated between -20° and 20°, shifted vertically or horizontally by 10% or zoomed by 30% [45]. The same augmentations were used for both tested models. The colour channels were scaled as required by the models, this is also known as data normalization. Images for VGG19 were set to [0, 1] and to [-1, 1] for MobileNetV3-Large.

Initially 10% of the dataset was set aside for testing the model later, no augmentation was performed on this set. Then, the remaining 90% of the dataset was randomly split, 80% of which was used for training the model and 20% was used for validation purposes during training. Therefore no images seen by the model during the training phase were later used for evaluation.

### Model architecture

**Fig 3:**
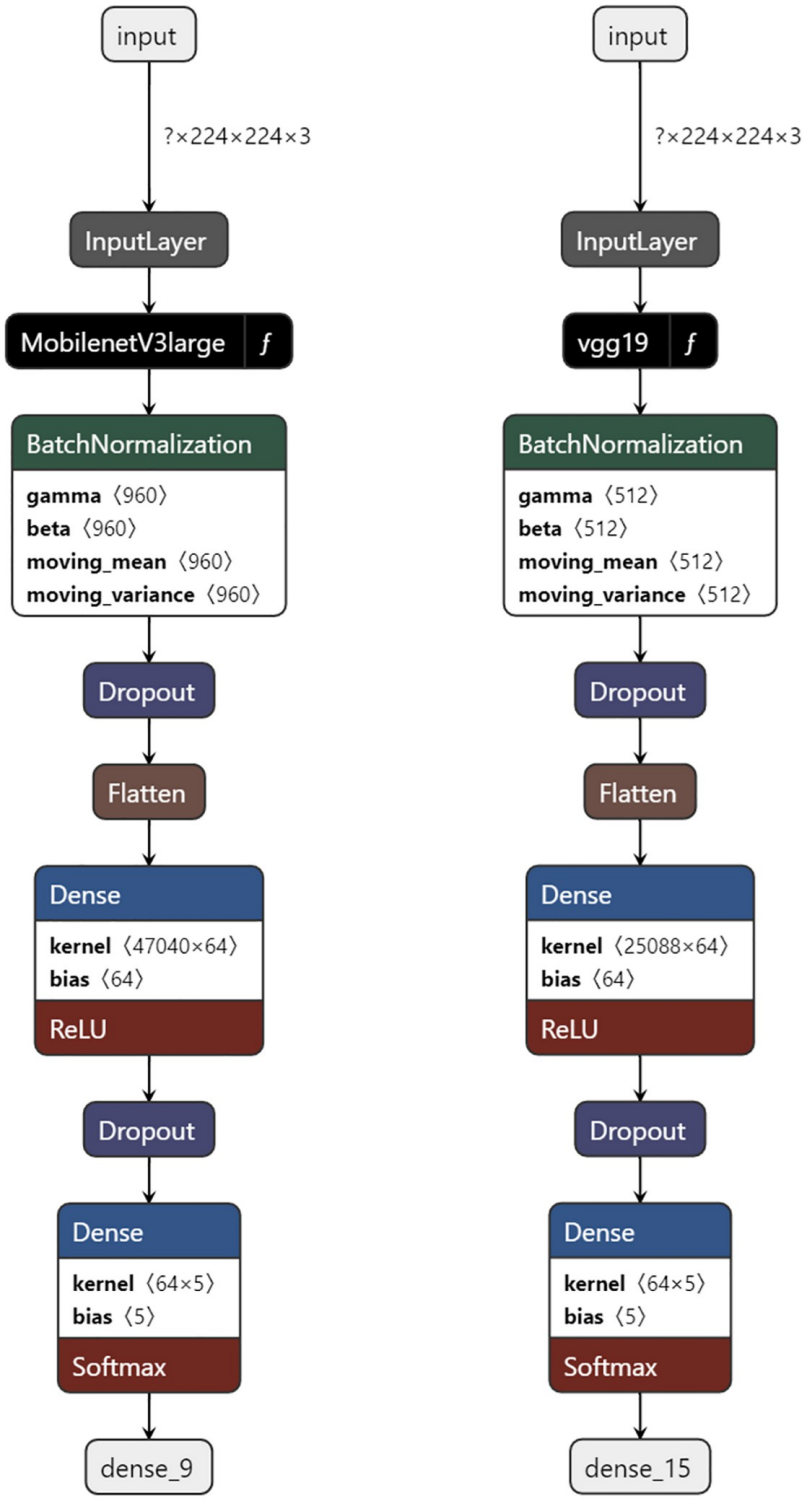
Flowchart of the architectures. For both models using MobilenetV3-Large and VGG19.

Two deep learning models were tested, VGG19 and MobileNetV3-Large [46, 47]. They were used with weights and biases pre-trained on the ImageNet visual recognition dataset [48]. VGG19 was selected as a benchmark model and the MobileNet variant was selected for its small size (MB), which could be useful in a potential application case using drones or other mobile devices. Both of our models use the same architecture. It starts with the respective pre-trained models directly after the input layer. Next, batch normalization is used to make training faster and more stable [49]. We applied two dropout layers, each dropping 40% of the input units, to avoid overfitting [50]. The inputs are flattened before going through the two additional densely connected layers. The first has a 64 dimension output space, using the ReLU activation function. The next dense layer gives us our output vectors with five dimensions, representing the five types of flies, this one uses a softmax activation function. A flowchart of the architecture for both models can be seen in Fig 3.

Both models were constructed, trained and tested using the Keras deep learning API on a NVIDIA Tesla T4 GPU. The training procedure differed between the pre-trained base models. VGG19 used the Adam optimizer, while for the MobileNet model the SGD optimizer was used to improve generalization [51]. For VGG19, the added top layers were trained for ten epochs, while the weights of VGG19 were frozen. Then VGG19 was unlocked and the whole model was fine-tuned in a second training run with a low learning rate of 0.00001 for an additional 20 epochs. With the MobileNetV3-Large base model, the training process only included 15 epochs, while the base model was frozen. Here, no fine-tuning was required.

### Evaluation method

For the validation of the models, different methods were used. First, a K-fold cross-validation is performed to obtain a reliable performance reading for each model. The data is split into k-equal parts. In our case a k of five was used. Now the model is trained on k minus one parts of the data and the validation is performed with the remaining share. This is repeated for all k parts, giving us five accuracy and loss values for each model. The overall model accuracy is obtained by averaging the results [52].

To represent the generalization of the models, a confusion matrix is used, as well as the precision, recall, and F1-score of each class. A confusion matrix provides a detailed statistical comparison of ground-truth labels and predictions. Its rows correspond to the expected outcomes, and the columns show what was predicted. The F1-score is a metric that is used to measure a model’s performance by combining both precision and recall. Precision measures how many of the positive predictions made are correct true positives, while recall measures how many of the positive cases the classifier correctly predicted over all positive cases in the data. The F1-score is calculated as the harmonic mean of both precision and recall [53].

## Results

### MobileNetV3-Large model

The MobileNetV3-Large model was trained using 5-fold cross-validations with 15 epochs per fold and a batch size of 32, each fold was tested with a batch size of 10. Due to immense overfitting when fine-tuning was tested, no additional tuning was performed. The performance of the model was evaluated using a confusion matrix and four performance indicators: accuracy, precision, recall, and F1-score.

**Fig 4:**
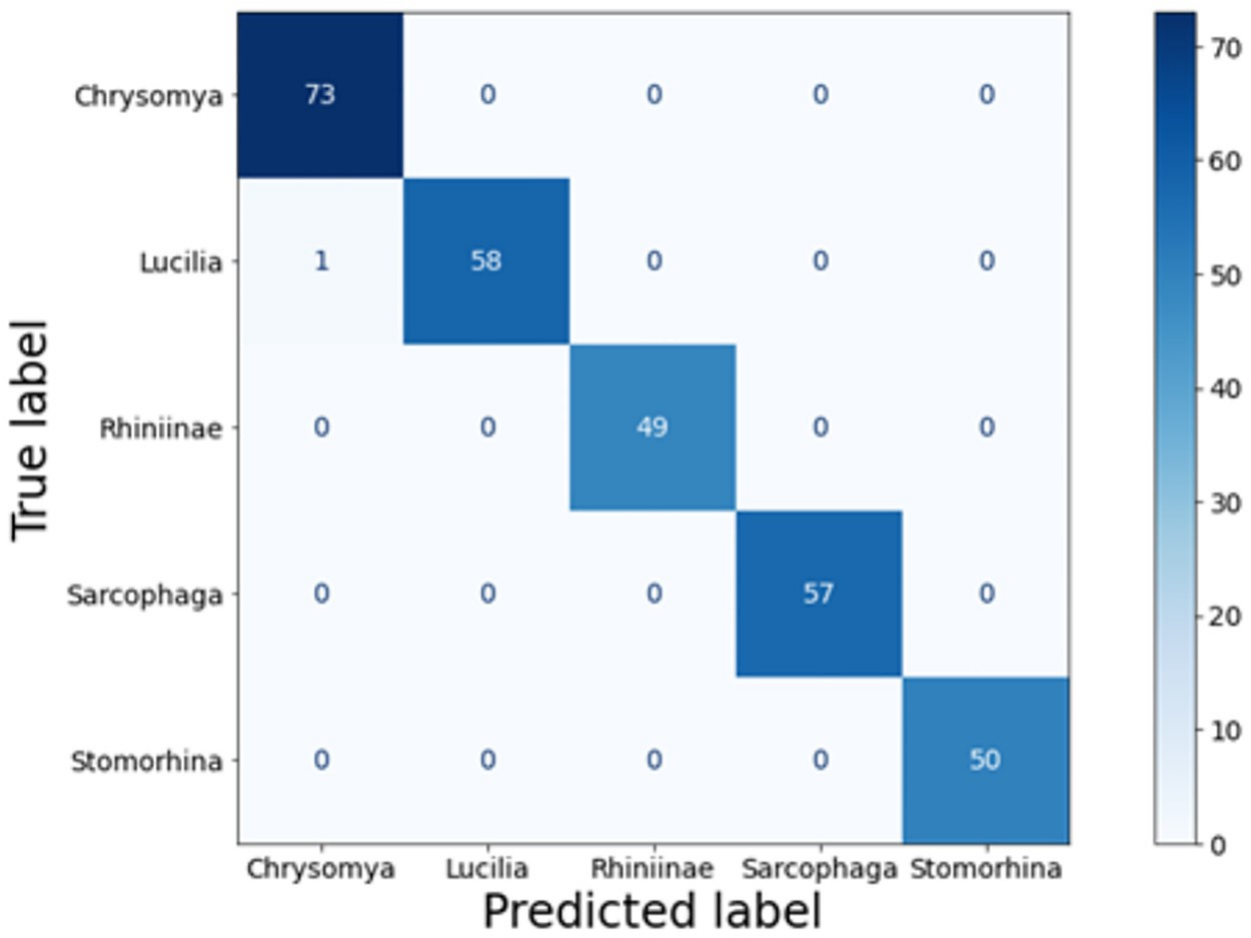
Loss and accuracy MobileNetV3-Large.

**Fig 5:**
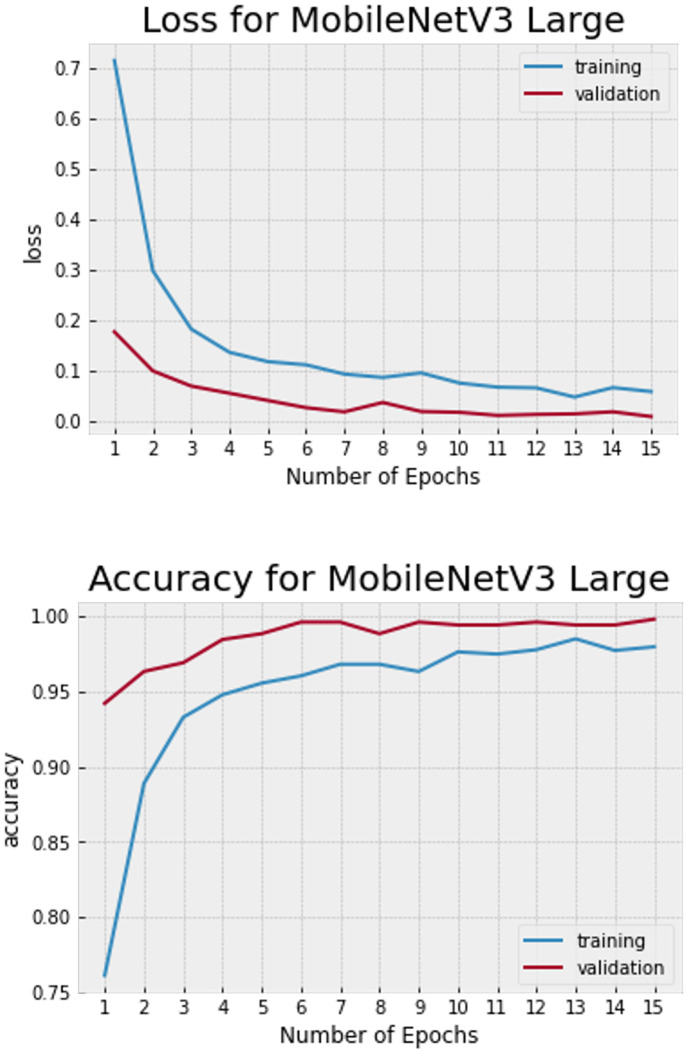
Confusion matrix MobileNetV3-Large.

The model achieved an accuracy of 99.65% in the first fold and 100% in the other four folds, resulting in an average accuracy of 99.93% and an average loss of 0.28%. The precision, recall, and F1-score for each fly species were 100%, indicating excellent performance (Fig 4). The confusion matrix, presented in Fig 5, provides a visual representation of the model’s classification results. The blue squares along the diagonal indicate correct classifications, while squares above and below the diagonal represent misclassifications [54]. Only one image of the genus *Lucilia* was misclassified, the other test images were assigned to the correct fly species.

The average time per step during testing for the MobilenetV3-Large model is approximately 1.036 seconds. This means that for each input batch during inference, the model needs a little more than one second to make a prediction [55].

### VGG19 model

**Fig 6:**
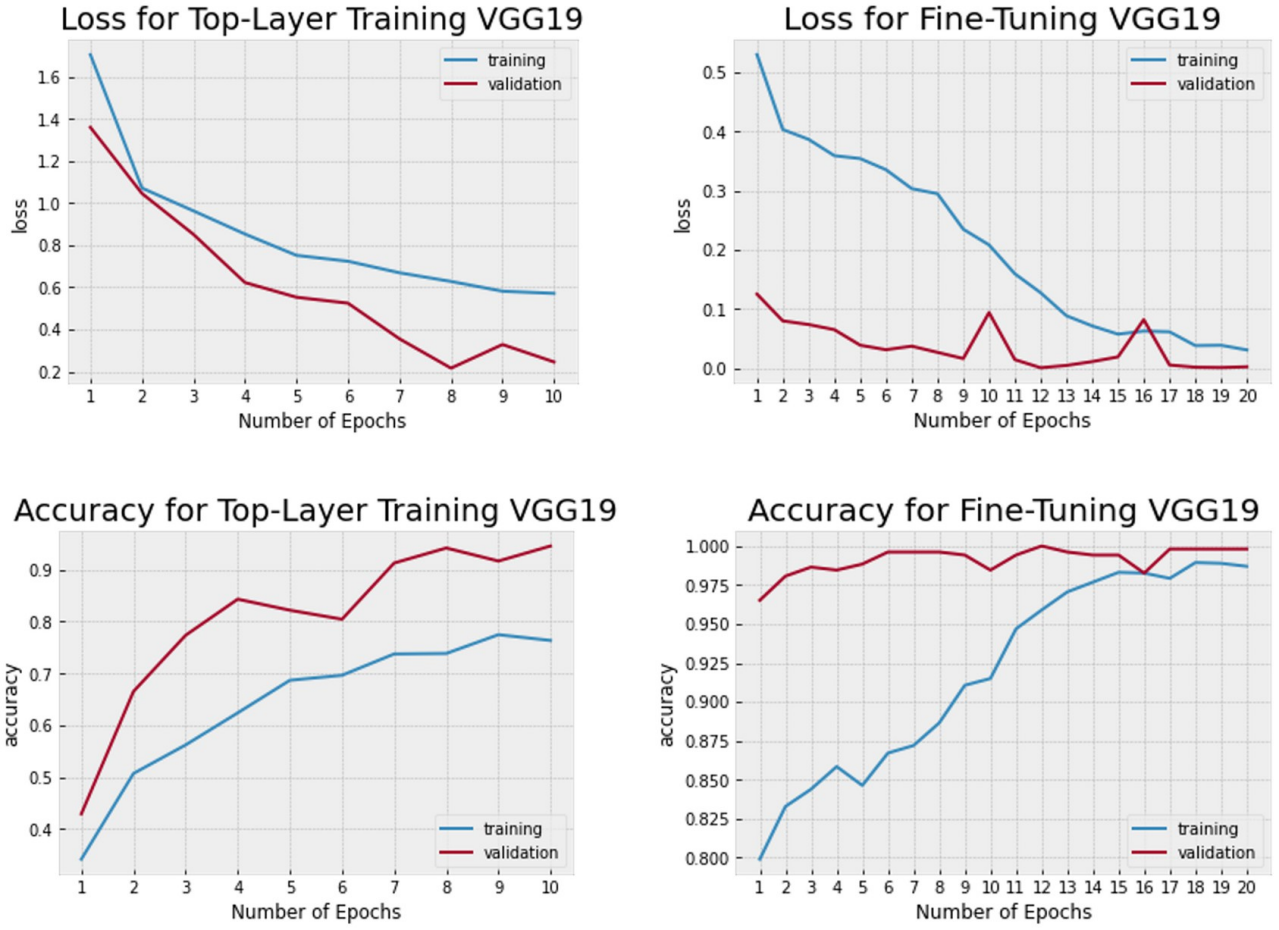
Loss and accuracy VGG19 before and after fine tuning.

**Fig 7:**
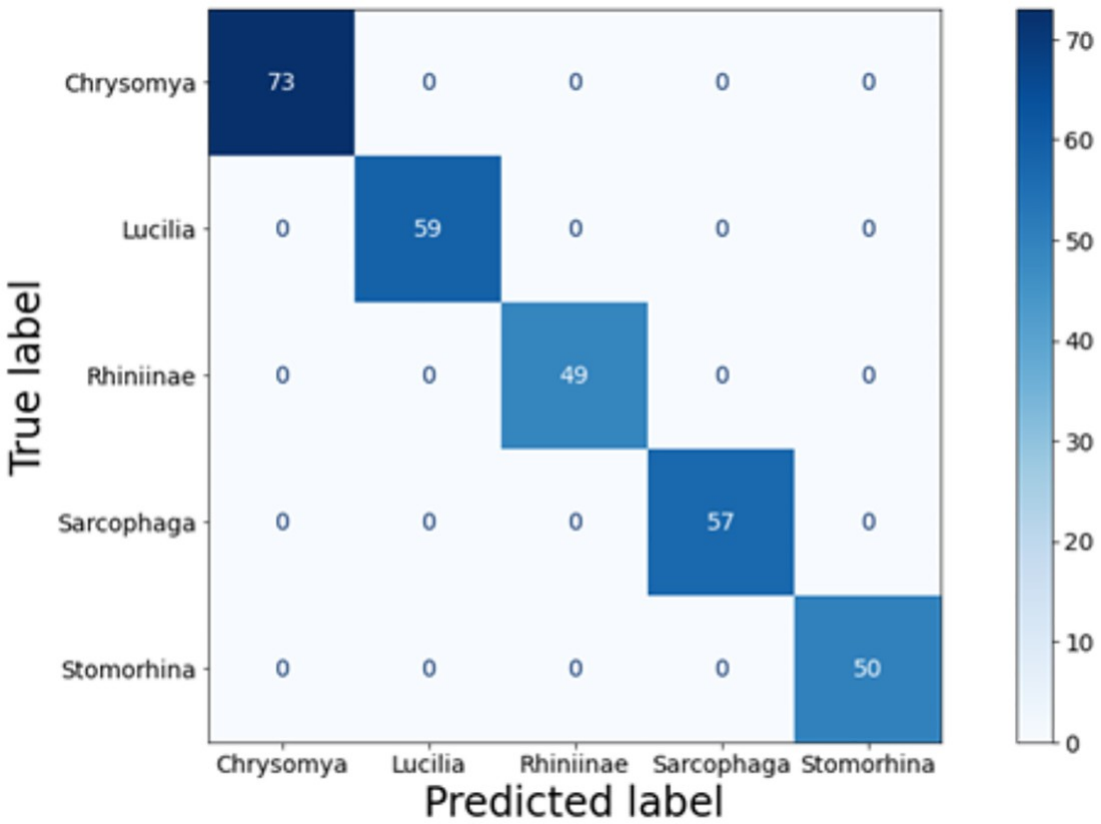
Confusion matrix VGG19.

In order to compare and potentially improve the results, a VGG19 model was also trained with the k-fold cross-validation method with 5 folds. The model was trained for 10 epochs, freezing all layers of VGG19. This initial training allowed the model to learn the general features of the dataset [56]. After the initial training, the remaining layers were unlocked and the model was fine-tuned with 20 additional epochs. During fine-tuning, the network adapts the learned features and improves the accuracy of the model. In our case, this resulted in higher accuracy and not overfitting as with the MobileNetV3-Large model (Fig 6). When evaluating the models performance on the validation data, it was found that the third fold demonstrated an accuracy of 98.95%, while the other four folds reached an accuracy of 100%. Therefore an average accuracy of 99.79% and a average loss of 1.36% was achieved over all 5 folds. This indicates that the model is able to achieve high accuracy while keeping its loss low. This also can be observed while watching the Confusion Matrix (Fig 7). All tested images were predicted correctly. All other calculated performance indicators namely precision, recall, and f1-score all reached 100%. For the VGG19 model the average time per batch during classification on the test dataset is 2.066 seconds.

In addition to the performance indicators the VGG19 model has significantly more parameters with a total of 21,632,453, out of which 1,607,045 are trainable in the first run and 20,025,408 are non trainable. Whereas MobileNetV3-Large has only a total of 6,011,141 parameters, out of which 3,012,869 are trained. This also results in a considerably larger file size of the saved VGG19 variant (253.6 MB) over the MobileNet alternative (35.6 MB).

## Discussion

Both the VGG19 and MobileNetV3-Large models provide high performance in the classification of the forensically important flies *Chrysomya, Lucilia, Sarcophaga, Rhiniinae*, and *Stomorhina*. In addition, the confusion matrix shows that all species have been correctly classified despite one fly out of 56 from the genus *Lucilia* being misclassified by the MobileNetV3-Large model. One possible reason for this could be that for each genus five different variants were included in the dataset [11] and it may be possible that, for example, a pigment adaption as an answer to stress can cause an assignment to another genera [57].

The related work shows that a CNN approach enables a high accuracy in the classification of insects, especially maggots [20]. But with an average accuracy of 97.33%, our models achieve a higher accuracy of over 99.70% each. So even though forensic entomology more often relies on maggots during the more detailed autopsy [58, 59] in case of the described problems in areas which were hard to access, the use of our model can make a crucial difference, particularly in finding and estimating PMI. This way it can be assessed whether any living people may still be found in debris, if flies are spotted who colonize bodies in the first few hours like *Calliphoridae* [25].

However, when selecting a neural network model for a particular task like the classification of images, the number of trained and non-trained parameters is an important factor [60]. Dense matrix multiplications and convolutions are the reasons for a high number of parameters [60] which make the use less suitable for applications with limited computational resources, such as mobile devices [47]. Therefore, less memory-intensive architectures should be used for embedded image processing applications [60, 61]. On the other hand, these smaller model architectures often are second to others like the VGG19 model in complex tasks [62].

The two trained models have a difference of 15,621,312 parameters and the files differ in a size by 218 MB. In addition, the inference time of a model is also an important metric to consider, especially for real-time applications where it is important that predictions are made quickly. The faster a model can make predictions, the better it can respond to changing input conditions [63]. Due to the results presented the prediction with the MobileNetV3-Large model is 1.03 seconds faster per batch than with the VGG19 model, which means it only needs half of the time to process.

During a study by Zhou et al. [55] MobileNetV2, quantized MobileNetV2, InceptionV3 and quantized InceptionV3 were used to process images on a smartphone. The time needed ranges from 0.103 seconds to 1.085 seconds and correlates directly with the model size. The InceptionV3 model, for example, needs 96,1 MB and the processing took 1.085 seconds. Whereas the quantized MobileNetV2 only has a size of 4,5 MB and processed the image within 0.103 seconds. Due to this study it can be concluded that our models especially the MobileNetV3-Large with 35,6 MB and 1.036 seconds to process one ten images batch fits in with the current state of science. The comparison also shows that faster times are possible through smaller architectures [55].

MobileNetV3-Large is optimized for efficiency and speed and is designed specifically for the use on mobile and embedded devices [47]. Taking into account the specific requirements and constraints of the application the balance between model complexity and performance is crucial. Regarding the huge difference of parameters of the two trained models and looking at the almost identical high accuracy both models achieve in classifying forensically important flies, the MobileNetV3-Large model may be better suited for tasks such as assisting pathologists during forensic investigations or rescue teams searching for bodies in crisis areas using camera-equipped drones. The speed of prediction that the MobileNetV3-Large model provides also can make a real-time application possible.

This is supported by the results of Qian et al. who compared the performance of popular models using different image datasets [64]. They found that MobileNetV3-Large provides high-quality classification for images with clearly identifiable objects. The resolution of the images is not critical and good results can be achieved even at low resolution. A large training data set with frequently occurring objects and typical shapes and states, as well as frequently occurring backgrounds is advantageous, but the differences between object categories don’t need to be obvious, as MobileNetV3 can accurately capture the features according to Qian et al.

Nevertheless, there is still the question whether drones equipped with the MobileNetV3-Large model can actually help solving the described problem. For this, exact information on the PMI must be provided using forensically important flies in areas that are difficult to access. It is questionable whether the same excellent results can actually be obtained under real conditions and at different distances, or whether, for example, unfavorable weather conditions will affect the accuracy.

A solution to this problem may be to add sensors to the drone that measure the perfect distance between a fly and the drone to get the best possible pictures. In addition, the drone can be equipped with GPS to geotag images and analyze them later in a following step [23]. For this purpose, the VGG19 model can also be used.

Despite this, it should be said that the model can be used in research and development for a first integration step to search for bodies because even if the PMI is estimated incorrectly, it supports rescue forces in recovering bodies in the right areas. This would counteract the problem that there is currently an acute shortage of skilled workers. This would reduce costs and could make an important contribution to the further development of rescue and recovery work.

## Conclusion

We have developed two highly accurate classification models, achieving almost 100% accuracy using two CNN models, the MobileNetV3Large and the VGG19 architecture. These trained models demonstrate that a CNN and a MobileNetV3-Large network are promising approaches to classifying forensically important flies. The dataset includes images of different fly genera that were taken from two different heights, a superior and a lateral position [11]. In addition, data augmentation was used to address a lack of individual data and dataset imbalances.

This work is one of the first attempts to use images of forensically important flies to estimate the PMI of victims and to assist rescue teams. It achieved higher accuracy than the work of Apasrawirote et al., in which the maggots of forensically important flies were classified [20].

Both models can classify five different genera (*Chrysomya, Lucilia, Sarcophage, Rhiniinae, Stomorhina*), including five specimens of each genus. The internal validity of our models are high due to the use of train-test-validation splitting, where testing was carried out on unseen and not augmented test data [52, 65]. All images were correctly classified in testing, except for one image by the MobileNetV3-Large architecture model.

The VGG19 and MobileNetV3-Large models are relevant to the practice because it is difficult to locate bodies in crisis areas, there is a shortage of skilled workers, and autopsy processes are time consuming. The models represent an important first step in utilizing machine learning for forensic entomology.

### Limitations

The main limitation of this work is based on the accurate data collection [11]. Blurred and low-quality images were removed from the dataset we worked with, that is why the dataset of genus images was imbalanced and leads to the limitation, that the images of the flies from the crime scene also need to be high-quality. Another limitation based on the dataset we used is that the specimens are labeled just to the genus level (*Chrysomya, Lucilia, Sarcophago, Rhiniinae, Stomorhina*). The individual species are correctly assigned and labeled, but at the species level only the genus was labeled. Therefore, research questions that require species-level identification may have difficulties in more specific classifications.

Moreover forensic entomology includes not only the fly that were part of the dataset. Next to *Callipohoridae, Sarcophagidae* and *Rhiniidae* also *Muscidae, Piophilidae, Syrphidae* and *Otitidae* are forensically important fly genus that are not represented [25] in our dataset.

In addition, a possible limitation might be that we only used the splitted image data and in the end also some individual external images from the internet to validate the performance but no complete external dataset.

Moreover, the flies of the dataset were already dead when photographed [11]. At this point, despite very good storage conditions, slightly altered appearances may occur [57], for example, the eyes may dry out and become cloudy or discolored. This may be a reason why the high accuracy of the built model can be lower in the real world. The acquisition of additional data from natural environments could further enhance the accuracy and prediction performance of the model.

### Future work

For the implication of drones finding bodies and estimating the PMI, future work can investigate potential limitations, for example, the models accuracy in different distances using a drone in real life or the effect of bad weather conditions. Doing this, it may be useful to develop design strategies to perfectly combine the model with the drone. The flight path or the quality of the images are important factors that should be coordinated.

It also may be an opportunity to test the model in real-world scenarios, for example, for search- and recovery missions. During this, efficiency can be assessed and potential improvements can be identified, so that the model can be implemented one day.

Due to the fact that the dataset consists of laboratory images, future work can train our model on images from real-world scenarios, where the flies are e.g. still alive. With this extension of the dataset the real-life application could be more successful.

Another possibility for future research can also be the development of more memory-efficient architectures for embedded image processing applications than the MobileNetV3-Large model. Compared to the VGG19 model, it provides a lighter architecture, is suitable for devices with limited resources, and shows higher training efficiency and speed [47, 62]. However, the choice of model ultimately depends on a variety of factors, including the specific requirements of the application, the size of the data set, and the available hardware. That is why it is important to improve the inference time to actually be able to implement machine learning approaches to the described problem. One possibility for a future work could be training the MobileNetV3-Small, which in addition to the MobileNetV3-Large was also presented by Howard et al. and is suitable for low resource applications [47]. But nevertheless our excellent performance results make an important contribution so that a lighter architecture should not compromise good results.
